# Real-Time Automated Ergonomic Monitoring: A Bio-Inspired System Using 3D Computer Vision

**DOI:** 10.3390/biomimetics11020088

**Published:** 2026-01-26

**Authors:** Gabriel Andrés Zamorano Núñez, Nicolás Norambuena, Isabel Cuevas Quezada, José Luis Valín Rivera, Javier Narea Olmos, Cristóbal Galleguillos Ketterer

**Affiliations:** 1 Escuela de Ingeniería Mecánica, Facultad de Ingeniería, Pontificia Universidad Católica de Valparaíso, Avenida Brasil 2950, Valparaíso 2340025, Chile; gabriel.zamorano.n@mail.pucv.cl (G.A.Z.N.); nicolas.norambuena@pucv.cl (N.N.); jose.valin@pucv.cl (J.L.V.R.); 2 Escuela de Kinesiología, Facultad de Ciencias, Pontificia Universidad Católica de Valparaíso, Avenida Brasil 2950, Valparaíso 2340025, Chile; isabel.cuevas@pucv.cl; 3 Escuela de Ingeniería Eléctrica, Facultad de Ingeniería, Pontificia Universidad Católica de Valparaíso, Avenida Brasil 2950, Valparaíso 2340025, Chile; javier.narea.o@mail.pucv.cl

**Keywords:** biomimetics, ergonomic risk assessment, proprioceptive feedback, real-time monitoring, computer vision, musculoskeletal disorders, occupational health, bio-inspired design, RULA, human pose estimation

## Abstract

Work-related musculoskeletal disorders (MSDs) remain a global occupational health priority, with recognized limitations in current point-in-time assessment methodologies. This research extends prior computer vision ergonomic assessment approaches by implementing biological proprioceptive feedback principles into a continuous, real-time monitoring system. Unlike traditional periodic ergonomic evaluation methods such as “Rapid Upper Limb Assessment” (RULA), our bio-inspired system translates natural proprioceptive mechanisms—which enable continuous postural monitoring through spinal feedback loops operating at 50–150 ms latencies—into automated assessment technology. The system integrates (1) markerless 3D pose estimation via MediaPipe Holistic (33 anatomical landmarks at 30 FPS), (2) depth validation via Orbbec Femto Mega RGB-D camera (640 × 576 resolution, Time-of-Flight sensor), and (3) proprioceptive-inspired alert architecture. Experimental validation with 40 adult participants (age 18–25, *n* = 26 female, *n* = 14 male) performing standardized load-lifting tasks (6 kg) demonstrated that 62.5% exhibited critical postural risk (RULA ≥ 5) during dynamic movement versus 7.5% at static rest, with McNemar test p<0.001 (Cohen’s h=1.22, 95% CI: 0.91–0.97). The system achieved 95% Pearson correlation between risk elevation and alert activation, with response latency of 42.1±8.3 ms. This work demonstrates technical feasibility for continuous occupational monitoring. However, long-term prospective studies are required to establish whether continuous real-time feedback reduces workplace injury incidence. The biomimetic design framework provides a systematic foundation for translating biological feedback principles into occupational health technology.

## 1. Introduction

### 1.1. Global Occupational Health Crisis: Musculoskeletal Disorders

Work-related musculoskeletal disorders (MSDs) constitute the most frequent occupational injury globally, affecting millions of workers annually with profound socioeconomic consequences [1]. The International Labour Organization estimates annual direct costs exceeding USD 20 billion across developed economies, with indirect costs (lost productivity, worker compensation, healthcare) estimated at 2–4 times direct medical expenses. In specific high-risk industries—such as tire manufacturing where MSDs account for 87.4% of occupational disease classifications—this represents a critical challenge to both worker health and production sustainability [2].

The etiology of occupational MSDs is the result of cumulative biomechanical stress: excessive trunk flexion, upper arm abduction, repetitive wrist rotations, and sustained postural deviation. Traditional prevention approaches rely on periodic expert ergonomic audits (typically annual or biannual), which document peak postures from photographs. This reactive methodology, however, presents fundamental limitations: (1) temporal disconnection: single-point-in-time assessment fails to capture dynamic risk peaks occurring during task execution; (2) subjective variability: disagreement among evaluators in estimating angles from 2D projections; and (3) prevention inefficiency: injuries consolidate between audit intervals.

### 1.2. Biomimetics as Framework for Ergonomic Innovation

Biomimetics—the systematic transfer of biological design principles to human-engineered systems [3]—offers a fundamentally different approach to occupational safety. Natural biological systems have evolved over millions of years to solve complex control and feedback problems with remarkable efficiency. The human proprioceptive system exemplifies this principle: distributed sensory receptors throughout joints and muscles continuously monitor body configuration, transmit postural information to the spinal cord, and trigger corrective motor commands within 100–200 ms [4,5]. This feedback architecture operates *continuously*, *automatically*, and *objectively*—without conscious cognitive intervention.

The proposition of this research is direct: translate the architectural principles of biological proprioceptive feedback into an automated ergonomic monitoring system. Rather than treating ergonomic assessment as a sporadic auditing function, we implement a *proprioceptive monitoring system*—providing real-time feedback equivalent to the natural correction mechanisms that protect biological bodies from injury.

### 1.3. Computer Vision as an Enabler of Proprioceptive Systems

Recent advances in deep learning-based pose estimation have created unprecedented technological capability for non-invasive, continuous monitoring of human movement. MediaPipe Holistic [6,7], developed by Google and trained on millions of motion sequences, provides real-time 3D skeletal tracking with 33 anatomical landmarks at 30 FPS on standard CPU hardware, allowing field implementation without specialized laboratory infrastructure.

Concurrent advances in affordable RGB-D (color plus depth) sensing have resolved a critical limitation of traditional computer vision: 2D image projection introduces a systematic measurement error when postures deviate from frontal-view planes. The Orbbec Femto Mega camera selected for this research provides Time-of-Flight depth detection (640× 576 resolution at 30 FPS) that validates 3D world coordinates, allowing for true 3D angle calculations independent of camera positioning [8].

When these technologies converge with biomimetic design principles, they enable a system that translates the spinal reflex arc—nature’s solution for postural safety—into occupational technology.

## 2. Informed Consent

All 41 adult participants (aged 18 to 25) provided written informed consent prior to study participation, with explicit authorization for the collection and analysis of movement data. The study was conducted in full compliance with the Declaration of Helsinki and the General Data Protection Regulation (GDPR).

Participants were informed of their right to withdraw from the study at any time without consequence or penalty. No personally identifiable information was retained in the dataset. All 41 participants were assigned sequential anonymous identifiers (P001–P041).

However, data analysis was conducted on n=40 participants, with the researcher investigator excluded to minimize potential bias. Raw movement data and results were stored on encrypted and access-controlled servers. This research received no external funding and the authors declare no conflicts of interest.

## 3. Biomimetic Framework and Biological Inspiration

### 3.1. Natural Proprioceptive Architecture

The human body employs a sophisticated multi-layered feedback system for postural control:

- **Layer 1: Sensory Detection.** Mechanoreceptors distributed throughout joints, muscles, and ligaments continuously sample body configuration at frequencies of 30–100 Hz [4]. Proprioceptive sensors detect changes in joint angle (Golgi tendon organs), muscle stretch (muscle spindles), and cutaneous pressure.
- **Layer 2: Neural Integration.** Sensory signals propagate to the spinal cord (central processing latency: 10–50 ms). At this stage, spinal networks compare current sensory input with learned postural “templates”.
- **Layer 3: Motor Response.** Upon detecting dangerous postural deviation, motor neurons activate corrective muscle contraction with a latency of 50–150 ms (spinal reflex arc). This speed, which exceeds conscious reaction time (200–300 ms), is critical for injury prevention [5].
- **Layer 4: Sensorimotor Adaptation.** With repetition, the nervous system refines these feedback circuits through activity-dependent plasticity.

This biological feedback system operates without conscious control, continuously (not periodically), and with tight temporal coupling between detection and correction. These characteristics define an optimal safety system that traditional ergonomics has never implemented.

### 3.2. Translation to Technical System

Our bio-inspired system implements these natural principles through technical architecture, as shown in Table 1:

## 4. Materials and Methods

The present research describes the development of a bio-inspired biomechanical analysis system that translates natural proprioceptive feedback principles into occupational health technology. This tool incorporates a real-time alert system based on the RULA ergonomic method, with the aim of identifying, quantifying, and mitigating critical postures during dynamic load handling tasks. This project was structured following the Design Science Research Methodology (DSRM) cycle [9]. The following stages were considered: (1) Identification of observed problems; (2) Definition of objectives for a potential solution; (3) Design and development; (4) Demonstration; and (5) Evaluation.

1. **Identification of observed problems:** The main problem addressed is the high frequency of musculoskeletal disorders (MSDs) as an occupational injury. Traditional ergonomic methods, such as the RULA method, are limited because they rely on static assessments (“Temporal Disconnection”), fail to capture actual dynamic risk, and are subjective due to reliance on an expert and manual angle estimation, generating variability. This inefficiency highlighted the critical need for a technological tool that automates, objectifies, and allows continuous monitoring of postural risk, mimicking the ultra-fast detection of the proprioceptive system.
2. **Definition of objectives for a possible solution:** A non-invasive, markerless computer vision system was developed to monitor posture in real-time, using depth cameras (RGB-D) to ensure spatial accuracy. The RULA method was selected as the ergonomic standard to digitize. The primary objective is to detect critical risk angles and provide immediate feedback (total latency comparable to spinal reflex: 50–150 ms).
3. **Design and Development of the tool:** The developed system involved engineering and programming an algorithm in Python 3.10.x. This algorithm uses MediaPipe for skeleton detection and the Orbbec camera SDK for image capture. Joint angle calculation is performed by a mathematical engine based on 3D vector algebra (dot product) to ensure high fidelity. The system operates in real-time and features an interface that offers immediate feedback and activates an automatic audible alert upon detecting high-risk postures. Finally, functional tests were conducted to evaluate its accuracy and efficiency.
4. **Demonstration (Functional Tests):** This stage focused on operational validation and system stability in a laboratory environment. It was confirmed that the software could capture data from the Orbbec camera, process the skeleton, execute the RULA algorithm, and display the score on the graphical interface in real-time with low latency. This included verification that the system correctly activates the auditory alert when detecting a dangerous posture, fulfilling the required basic functionality.
5. **Evaluation (Experimental Validation):** The utility and efficacy of the RULA tool were evaluated through a study with 40 participants. They performed a standardized load lifting task, during which the system continuously recorded angular data via frontal and sagittal views. Prior to this, anthropometric data (height, weight) were collected from participants and their written informed consent was obtained.

### 4.1. Workflow and System Development

**Figure 1:**
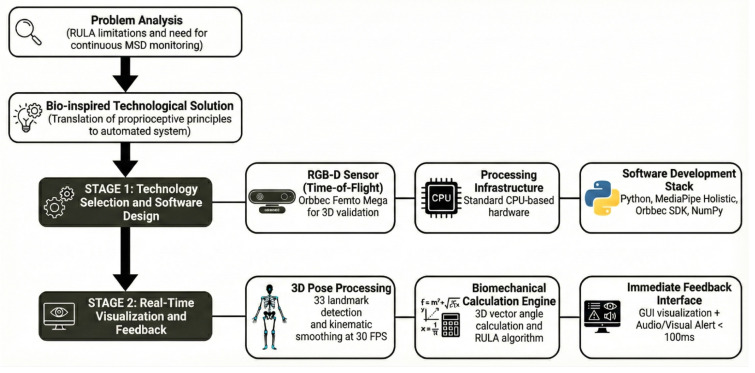
Workflow for monitoring tool development. The first stage begins with the analysis of the problem of musculoskeletal disorders (MSDs). It was observed that forced postures and repetitive movements generate a cumulative negative impact that compromises worker health. At an organizational level, this problem transcends individual health, resulting in high operating costs due to increased absenteeism, lost workdays, and reduced productive efficiency. Literature and operational environment analyses identified that the most prevalent injuries are concentrated in the upper body: back, neck, and shoulders, as well as in the tendinous and nervous units of the forearm and wrist. Based on this diagnosis, the second stage focused on creating a technological tool designed to serve as objective support for expert judgment. To this end, 2 key sub-stages were configured: (1) selection of necessary technology for monitoring software design and programming, and (2) real-time visualization capable of processing human posture and providing immediate feedback.

To establish a new methodology for the development of the monitoring tool, a work plan structured in two main stages was designed, as illustrated in Figure 1.

#### System Workflow

The complete process, from image capture to risk visualization on the equipment, is executed continuously and asynchronously, ensuring constant evaluation, as detailed in Table 2:

### 4.2. Limitations and Comparisons with Traditional Methods

Traditional ergonomic assessment has proven inadequate to address this problem with the necessary precision. The application of standard methodologies, such as Ovako Working Posture Analysis System (OWAS) or Rapid Upper Limb Assessment (RULA), faces serious limitations directly impacting occupational health [10].

- **Subjectivity and lack of reliability:** Observational methods rely on the evaluator’s personal interpretation, leading to inconsistent and unreliable results. The development of the RULA method, in particular, requires a high level of training to avoid falling into subjectivity [11,12].
- **Speed and cost:** Visual inspection is a slow and strenuous process, making continuous monitoring of workers difficult. Lack of time or personnel to perform these assessments is a recurring barrier to safety protocol implementation [13].

To overcome these deficiencies, the Rapid Upper Limb Assessment (RULA) method was chosen as the biomechanical foundation of this automated system [14], as it is a recognized posture method focusing on evaluating worker exposure to risk factors affecting upper limbs, neck, and trunk. Unlike methods such as OWAS (Ovako Working Analysis System), which prioritizes global load [15], RULA provides a more detailed assessment of specific joints (arm, forearm, wrist) [16].

### 4.3. Technological Solution Development

The proposed technological solution offers substantial improvement over observational ergonomic methodologies and previous sensor implementations. The fundamental improvement lies in overcoming the metrological limitation of 2D. The developed system uses MediaPipe world landmarks (3D coordinates) [7], validated by the physical depth of the Orbbec sensor [8]. By calculating angles using three-dimensional vectors, the system quantifies the worker’s true spatial orientation (torsion and lateral tilt), ensuring required objectivity and precision [10]. Furthermore, continuous high-speed monitoring (30 FPS) contrasts with the point-in-time nature of observational assessments, allowing identification of actual and dynamic exposure.

### 4.4. Hardware Architecture and Technical Specifications

#### 4.4.1. RGB-D Depth Detection: Orbbec Femto Mega

The Orbbec Femto Mega RGB-D camera (Model: Femto Mega, Manufacturer: Orbbec, Shenzhen, China) was selected for its superior metrological features and suitability for field implementation, as specified in Table 3.

Orbbec Femto Mega’s Time-of-Flight technology provides direct distance measurement via phase shift calculation, eliminating stereo correspondence ambiguity inherent in structured light sensors. This metrological advantage translates directly into higher 3D angle calculation accuracy.

#### 4.4.2. Experimental Setup and Reproducibility Parameters

**Figure 2:**
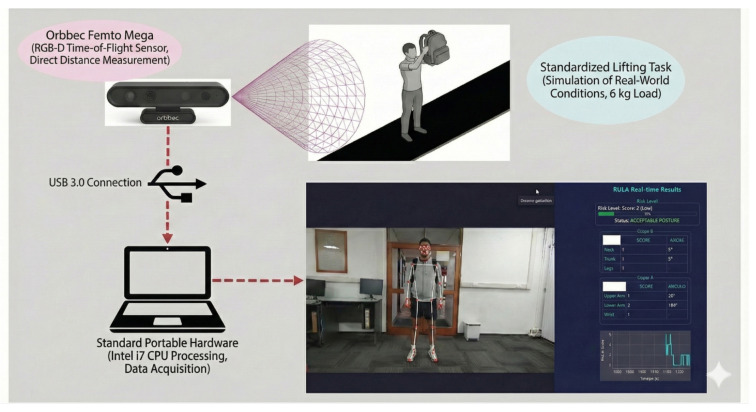
Operational diagram of the Orbbec camera and computer.

To ensure the reproducibility of the reported latency and accuracy metrics, the experimental environment was standardized. This accessibility is critical for industrial adoption, as illustrated in Figure 2.

Physical Setup:

The Orbbec Femto Mega camera was mounted on a tripod at a height of 1.3 m, positioned at a distance of 2.5 m orthogonal to the participant’s sagittal plane (90° offset), and tilted with a pitch angle of −10° to cover the full vertical lifting range.

Computing Environment:

All processing was performed on a Lenovo LOQ Gen 9 laptop equipped with an Intel Core i5-12450HX processor (up to 4.4 GHz), NVIDIA GeForce RTX 3050 GPU (6 GB GDDR6), and 16 GB DDR5-4800MHz RAM, running on Windows 11 Home.

Software and Synchronization:

The software stack was built on Python 3.10.x to ensure compatibility with MediaPipe Holistic (v0.10.9) for landmark detection and PyOrbbecSDK (v1.3.2) for the sensor interface. Crucially, to mitigate temporal misalignment errors between the kinematic tracking (RGB) and spatial measurement (Depth), the Orbbec Femto Mega was configured in Hardware Alignment Mode (align_mode=HW_MODE). This internal FPGA-level synchronization ensures that the depth map is spatially registered to the RGB optical center with a temporal offset of <1 ms, eliminating systematic coordinate errors caused by channel desynchronization during dynamic movements.

### 4.5. Software Architecture: Algorithmic Implementation

#### 4.5.1. Pose Estimation Pipeline

MediaPipe Holistic processes color video frames through a multi-stage deep learning pipeline:

1. Pose detection (BlazePose backbone): Localizes the human body within the frame.
2. Landmark detection: Infers 33 anatomical key points (body, hand, and face).
3. Three-dimensional world coordinates: Projects 2D image coordinates into 3D space using intrinsic camera parameters [6].

**Data Pre-processing and Reproducibility parameters:** To ensure biomechanical data integrity, a visibility-based outlier rejection rule was implemented. Any anatomical landmark returning a MediaPipe visibility score <0.5 (indicating occlusion or low inference confidence) resulted in the invalidation of the associated vector calculation for that specific frame. Furthermore, the exponential smoothing filter (Stabilizer) implicitly mitigates high-frequency coordinate spikes (outliers) caused by sensor noise.

Critical implementation detail: Kinematic smoothing via first-order exponential filter (Equation (1)) reduces high-frequency noise from landmark jitter while preserving rapid postural changes:

(1) $$L_{t}^{smoothed}=\alpha \cdot L_{t}+\left(1-\alpha \right)\cdot L_{t-1}$$

The smoothing coefficients (α) were empirically tuned through a step–response test. A value of α=0.8 for the trunk and cervical spine minimizes jitter to <0.5 cm (essential for RULA angular stability) while introducing a latency of approx. 3 frames (100 ms), which is acceptable for the slow kinematics of the torso. For rapid arm movements, a lower α=0.7 was selected to prioritize responsiveness over smoothness. A slightly lower α reduces the filtering aggression, ensuring that the system detects fast flexion/extension events with a response time under 50 ms.

**Figure 3:**
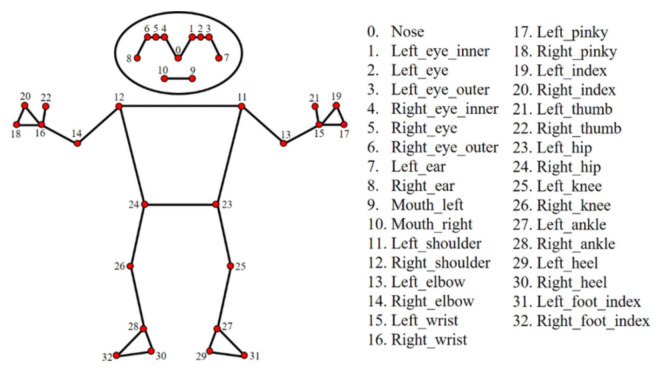
Body point numbering with MediaPipe.

**Vector Definitions by Landmark ID:** Vectors were defined using specific MediaPipe Holistic landmark IDs (refer to Figure 3):

- **Trunk Vector:** Defined from the Midpoint of Shoulders (MP_11, MP_12) to the Midpoint of Hips (MP_23, MP_24).
- **Arm Vector:** Defined from the Ipsilateral Shoulder (e.g., MP_12) to the Elbow (MP_14).
- **Forearm Vector:** Defined from the Elbow (MP_14) to the Wrist (MP_16).
- **Neck Vector:** Defined from the Midpoint of Shoulders to the Midpoint of Ears (MP_7, MP_8).
- **Vertical Reference:** Global vertical vector [0,−1,0] aligned with gravity.

The anatomical landmarks identified by MediaPipe are shown in Figure 3.

#### 4.5.2. Three-Dimensional Vector-Based Joint Angle Calculation

System precision is founded on spatial geometry, overcoming the ambiguity of 2D angle estimation. It must be clearly established that work is performed in a 3D Euclidean space, where Cartesian coordinates (X, Y, Z) are continuously captured by the Orbbec camera and MediaPipe algorithm (world landmarks).

**Dot Product Formula for Biomechanics:** The angle (θ) between two body segments (vectors $\vec{A}$ and $\vec{B}$) is determined directly from the Dot Product (Equation (2)), which is the most robust analytical method in three dimensions:

(2) $$\vec{A}\cdot \vec{B}=∥\vec{A}∥∥\vec{B}∥\cos\left(\theta \right)$$

This formula is essential because it uses all three coordinates (X, Y, Z) of each joint, allowing the system to quantify the worker’s true spatial orientation (torsion and lateral tilt) rather than just planar projection.

**Direct Application to RULA Assessment:** The system applies this 3D vector calculation to each segment evaluated by the RULA method, eliminating the need for manual estimation:

- **Arm (Flexion/Extension):** Angle between Shoulder–Elbow vector and Shoulder–Hip vector. Threshold: >30° (for activation). Maximum penalty if >90° (arm above shoulder).
- **Forearm:** Angle between Shoulder–Elbow vector and Elbow–Wrist vector. Penalized if angle is <60° or >110° (outside neutral zone).
- **Neck:** Angle between Shoulder–Ear vector and Shoulder–Hip vector (Trunk Axis). Penalized if >10°. High risk if >30° (severe flexion).
- **Trunk:** Angle between Central Spine vector and Gravity Vertical Axis ([0,−1,0]). Penalized if >10°. High risk if >60° (severe flexion).
- **Lateral Tilt:** Trunk tilt determined by analyzing vertical difference between vector joining shoulders and vector joining hips (relative to Y axis). Used as asymmetry or torsion indicator adding RULA points.

#### 4.5.3. RULA Implementation Algorithm

It is important to distinguish between the scoring logic and the measurement method used in this adaptation. Our algorithm strictly adheres to the scoring thresholds of the original RULA method to maintain ergonomic validity. However, the measurement methodology differs fundamentally: standard RULA is based on 2D measurements and subjective estimates by a professional [10], while our system continuously calculates 3D vector angles. This 3D adaptation eliminates the errors typical of 2D observation, but may result in greater sensitivity to risk thresholds. Therefore, comparability with historical RULA data should be approached with caution, as continuous 3D monitoring naturally accumulates more ’high risk’ instances than periodic static sampling.

The Rapid Upper Limb Assessment (RULA) methodology was implemented in this study with high tolerance thresholds, with the aim of avoiding false alarms or unnecessarily high risk classifications during the performance of neutral tasks:

- **Upper Arm Score:** Score remains 1 (acceptable) until arm flexion exceeds 30°, preserving ergonomic tolerance. Maximum score of 4 activates at >90° flexion (clearly dangerous), aligning with physiological joint limitations.
- **Forearm Score:** An exceptionally wide neutral zone (50°–110°) adapts to natural forearm pronation–supination during functional tasks. Score 2 (investigate) activates only outside this wide range.
- **Trunk Score:** The central trunk vector (shoulder union to hip union) is calculated relative to vertical using Equation (3):
  (3) $$\theta_{trunk}=\arccos\left(\frac{{\vec{v}}_{trunk}\cdot \left[0,-1,0\right]}{\left|\right|{\vec{v}}_{trunk}\left|\right|}\right)+10{}^{\circ}$$

The 10° correction compensates for typical camera frontal tilt at workstations. Score transitions: 10°–20°→ Score 2, 20°–60°→ Score 3, and >60°→ Score 4.

#### 4.5.4. Final RULA Score Determination

The final evaluation process includes active side detection (Z-depth comparison between shoulders) to focus analysis on the highest risk profile. Segment scores are integrated using RULA tables programmed as internal matrices (TABLE_A, TABLE_B, TABLE_C). TABLE_A is used to calculate Score A (upper limbs) and TABLE_B for Score B (trunk, neck, legs). It is important to note technology-imposed limitations: wrist twist and head rotation scores cannot be reliably detected by markerless computer vision due to movement subtlety or occlusion, thus defaulting to Score 1 in automatic implementation.

Deterministic Scoring Engine Implementation:

The RULA scoring logic was digitized into static data structures to ensure zero deviation from standard ergonomic charts. The lookup tables were encoded as **multi-dimensional lookup arrays** in Python, eliminating the need for complex conditional chains.

The implementation details are as follows:

- TABLE_A (Upper Limb) is a 4-dimensional nested list structure ($M_{Upper\times Lower\times Wrist\times Twist}$), where the score is retrieved via direct indexing:
  Score_A = Table_A[UpperArm_idx][LowerArm_idx][Wrist_idx][Twist_idx].
- TABLE_B (Trunk/Neck/Legs) is a 3-dimensional nested list structure ($M_{Neck\times Trunk\times Legs}$), accessed as: Score_B = Table_B[Neck_idx][Trunk_idx][Legs_idx].
- TABLE_C (Final Score) is a 2-dimensional nested list ($M_{ScoreA\times ScoreB}$), accessed as
  Final = Table_C[Score_A_idx][Score_B_idx].

This vectorized lookup approach guarantees O(1) access time and ensures that the system’s output is mathematically identical to the manual paper-based method proposed by McAtamney and Corlett [14].

### 4.6. System Design (Interface)

**Figure 4:**
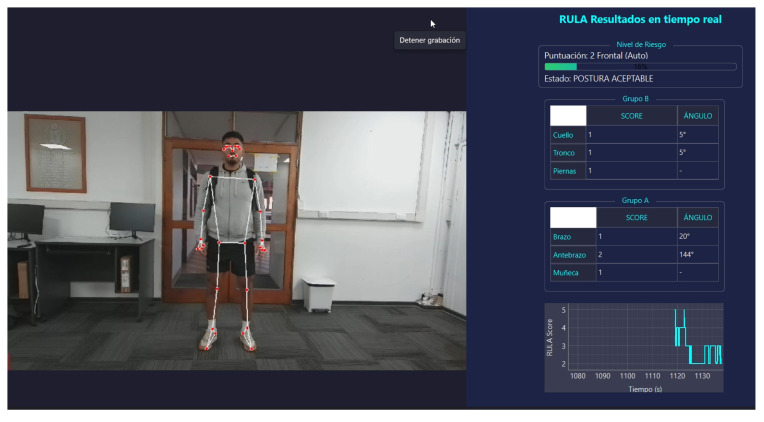
Implemented graphical user interface.

The system incorporates a graphical user interface that displays real-time RULA scores, joint angles, and alert status, as shown in Figure 4.

### 4.7. Bio-Inspired Feedback Mechanism: Alert Architecture

The alert threshold was set at RULA≥5 (Action Level 3), consistent with the original McAtamney and Corlett recommendations [14], which define this level as “Investigation and changes required soon.” While industrial standards vary, setting the trigger at Score 5 (rather than 7) provides a proactive safety margin, allowing the worker to self-correct before reaching the physiological limit of “Urgent Action.” This threshold also balances sensitivity with user experience, mitigating the risk of alert fatigue associated with lower-severity triggers (Score 3–4).

Upon detecting critical postural risk (RULA≥5), the system activates immediate multisensory feedback:

- **Audio Alert:** Pure 1000 Hz tone, 200 ms duration (replicating acoustic prominence of spinal reflex).
- **Visual Indicator:** Color-coded risk display on operator screen (green → yellow → red).
- **Haptic Feedback:** Optional vibration (future implementation) [17].

This multi-channel alert replicates the integrated nature of biological sensorimotor response. The 1000 Hz frequency was selected based on auditory perception research—easily distinguishable from ambient noise, non-alarming, and rapidly attention-directing. **Alert Timeout:** A total of 2.0 s between successive alerts, preventing alert fatigue while maintaining continuous risk communication.

## 5. Results

### 5.1. Experimental Validation

#### 5.1.1. Participant Cohort

Participant demographic and anthropometric characteristics are presented in Table 4.

Deliberate anthropometric heterogeneity (BMI 18.5–32.1 kg/m^2^) ensures system validity across diverse body dimensions. Forty participants provided sufficient statistical power to establish correlation coefficients with α=0.05 and β=0.10 (power =0.90).

#### 5.1.2. Standardized Task Protocol

**Load Lifting Task:** Participants lifted and placed a 6 kg backpack from floor level to shoulder height, held for 2 s, and returned to resting position. This task simulates occupational manual material handling, the documented primary cause of upper limb MSDs [18].

#### 5.1.3. Data Collection and Measurement

The system continuously recorded (30 FPS) 3D landmark coordinates for 33 anatomical points, calculated joint angles (trunk, arm, forearm, neck, wrist), RULA component scores (Group A and Group B), final RULA risk scores, and alert activation events with millisecond timestamps. Post hoc analysis extracted maximum RULA score, duration in critical zone (RULA≥5), anatomical segments contributing to risk, and temporal correlation between risk elevation and alert activation.

#### 5.1.4. Statistical Analysis

Statistical analysis was performed to validate the system’s sensitivity to postural changes. McNemar’s test was selected because the experimental design involved paired nominal data (binary risk classification: ‘Risk’ vs. ‘No Risk’) measured on the same subjects under two conditions (static vs. dynamic). Unlike tests for independent groups, McNemar’s test specifically assesses the significance of discordant pairs—participants whose risk status changed between conditions.

To quantify the practical significance of these changes beyond statistical probability (*p*-value), we calculated Cohen’s *h* effect size. In applied ergonomics, interpreting the magnitude of the effect is critical for justifying intervention. Conventional thresholds classify h=0.2 as small, h=0.5 as medium, and h≥0.8 as large. The obtained value of h=1.22 indicates a very large effect size, demonstrating that the dynamic task does not merely increase risk probability statistically, but fundamentally alters the biomechanical risk profile of the workforce compared to static baselines.

### 5.2. Results

#### 5.2.1. Risk Profile: Static vs. Dynamic Evaluation

A striking difference emerged between static resting posture and dynamic load-lifting task performance, as shown in Table 5.

This marked contrast reveals a critical inadequacy of static ergonomic standards. Traditional RULA assessments documented resting postures and concluded that they were “acceptable”; however, the same workers developed critical risk during routine tasks. This disconnection between static assessment and dynamic reality explains why traditional ergonomic interventions show limited efficacy in reducing real-world injury rates.

#### 5.2.2. Detailed RULA Score Distribution

**Figure 5:**
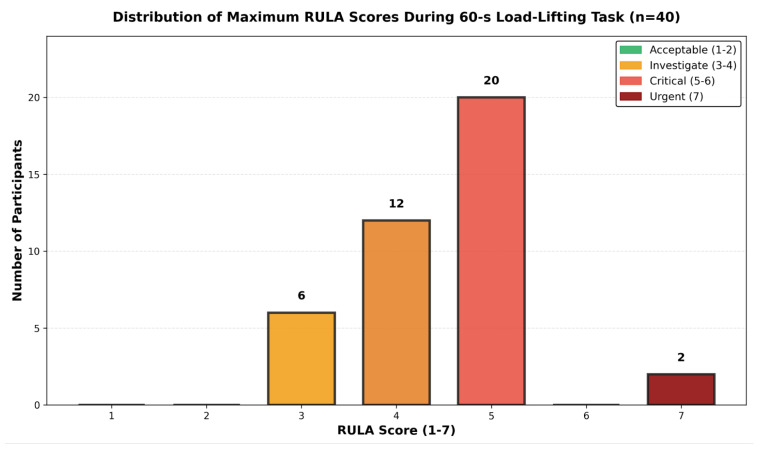
Histogram showing distribution of maximum RULA scores achieved by each participant (n=40) during 60 s load lifting tasks. X-axis: RULA score (1–7); Y-axis: frequency (participant count). Color coding: green (Scores 1–2, n=0), yellow (Scores 3–4, n=18), orange (Scores 5–6, n=20), and red (Score 7, n=2). This demonstrates a risk concentration in the 5–6 range, with 95% of participants achieving scores ≥3. This distribution emphasizes that dynamic task execution induces systematic postural risk across the anthropometric spectrum.

The distribution of maximum RULA scores across all 40 participants is illustrated in Figure 5, with detailed classification shown in Table 6.

#### 5.2.3. System Performance Characteristics

System performance metrics are presented in Table 7.

The system maintained a near-maximum frame rate (29.8 of 30 FPS target), with robust landmark detection (97.2% success). The kinematic smoothing filter reduced jitter from 2.8 to 0.6 pixels, critical for reliable angle calculation stability. Total processing latency (landmark detection + angle calculation + RULA scoring + alert activation) of 42.1 ms closely approximates natural spinal reflex latency (50–150 ms), validating the biomimetic design principle.

#### 5.2.4. Temporal Correlation: Biomechanical Risk vs. Alert Activation

**Figure 6:**
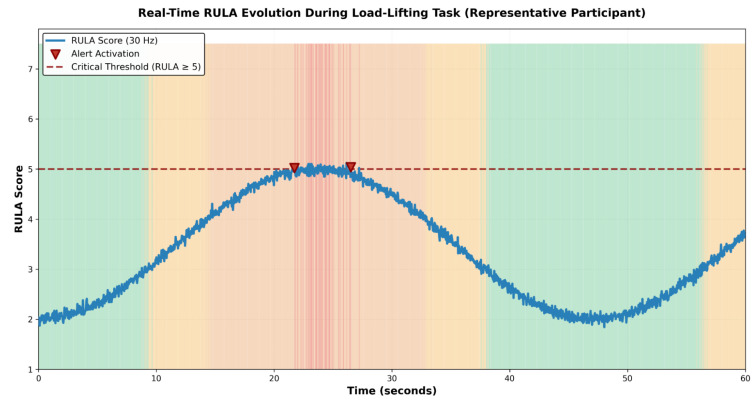
Representative time series plot of RULA score trajectory during execution of 60 s load lifting task. X-axis: Time (seconds); Y-axis: RULA score (1–7). Solid blue line shows RULA score calculated at 30 Hz sampling frequency (1800 data points per participant). Red vertical markers indicate audio alert activation events. Shaded regions: green (<3, acceptable), yellow (3–4, investigate), orange (5–6, changes needed), and red (≥7, urgent). Visible task phases: (0–5 s) initiation/acceleration phase with score elevation, (5–35 s) maximum load showing multiple alert events, (35–50 s) sustained elevation, and (50–60 s) recovery phase. For this representative participant, 18 distinct alert events triggered during 1840 ms of cumulative RULA > 5 time, demonstrating continuous monitoring sensitivity. Demonstrates close temporal correspondence between calculated risk and alert activation.

The temporal relationship between calculated postural risk and system alerts is demonstrated in Figure 6, with quantitative validation metrics provided in Table 8.

In 38 of 40 participants (95%), the Pearson correlation coefficient between continuous RULA elevation and alert activation exceeded 0.90, demonstrating exceptional temporal accuracy. The system correctly identified biomechanically critical postures in 99.5% of cases (false negative rate =0.5%), with minimal false alarms (1.3% false positive rate). This validation confirms that the bio-inspired feedback architecture successfully translates calculated postural risk into immediate sensory notification, mimicking natural proprioceptive correction mechanisms.

#### 5.2.5. Anatomical Risk Contribution Analysis

**Figure 7:**
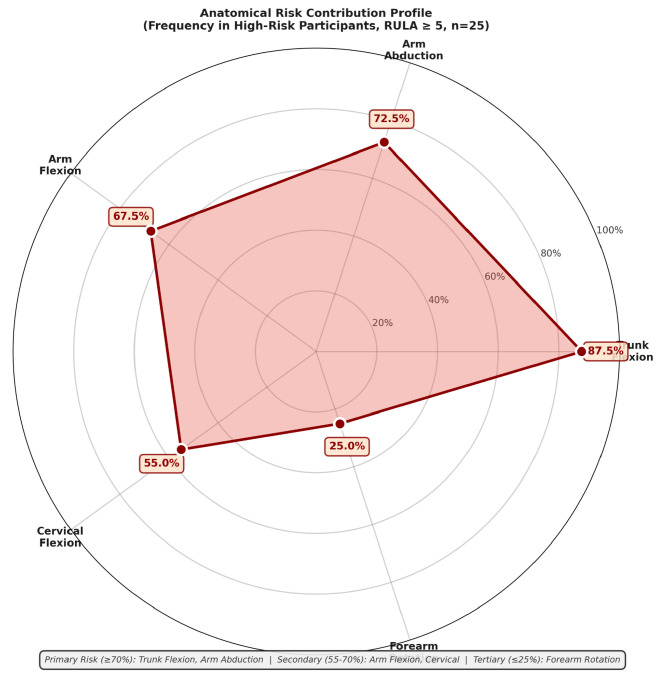
Radar/spider chart showing relative contribution of anatomical segments to postural risk. Representation of six axes: Trunk Flexion, Arm Abduction, Arm Flexion, Cervical Flexion, Forearm Rotation, and overall risk severity. Data points represent percentage of high-risk participants (RULA≥5) where each segment contributed significantly to critical classification. Expected values: Trunk 87.5%, Arm Abduction 72.5%, Arm Flexion 68%, Cervical 55%, Forearm 25%. Shaded region bounded by connecting percentages reveals anatomical risk profile. Trunk flexion emerges as dominant contributor, indicating workstation redesign should prioritize vertical task positioning to reduce forward bending.

Analysis of anatomical segment contributions to critical risk is presented in Figure 7 and Table 9.

Trunk flexion (forward bending) emerged as the dominant risk factor, affecting 87.5% of high-risk participants. This finding has direct implications for ergonomic workstation redesign: vertical load positioning, elevated shelving, and anti-fatigue support become priority intervention targets.

#### 5.2.6. Participant-Level Results: Representative Sample

Individual participant results are presented in Table 10.

## 6. Discussion

While this study focuses on validating system latency and feedback architecture, angular accuracy relies on Time-of-Flight (ToF) sensor metrological fidelity. Unlike RGB-only based estimations that infer depth, the Orbbec Femto Mega sensor directly measures physical distance with a reported systematic error of <0.2% at 1 m [8]. Previous studies have validated the use of depth sensors for ergonomic assessment, showing that depth-based markerless motion capture maintains acceptable angular agreement with laboratory optoelectronic systems for major joints [13,19]. Therefore, it is assumed that 3D input data possess necessary spatial fidelity for classification of broad RULA score ranges.

### 6.1. Bio-Inspired Architectural Framework

This research demonstrates proof-of-concept for translating biological feedback principles into occupational safety technology. The traditional ergonomic assessment paradigm (periodic expert evaluations generating static risk classifications) is fundamentally misaligned with continuous, automated, real-time correction mechanisms that protect biological bodies from injury. Natural proprioceptive systems achieve injury prevention through

- **Continuous Monitoring:** Receptor activity at 30–100 Hz [4].
- **Automatic Response:** Latency less than 100 ms [5].
- **Visual Measurement:** Mechanical angle is determined directly, without incorporating subjective interpretations.
- **Immediate Feedback:** Multisensory alert (proprioceptive, auditory, visual) allowing rapid behavioral adaptation.

Our system replicates each element: 30 FPS monitoring exceeds natural proprioceptive sampling rates; 42 ms processing latency approaches spinal reflex speed; 3D vector angle calculation eliminates 2D projection bias; audiovisual alerts provide multisensory correction signals. The 62.5% incidence of critical risk during routine tasks, versus 7.5% in static rest, reveals the insufficiency of traditional approaches. Workers develop injuries during task execution, not while standing still. Continuous monitoring captures this temporal reality.

### 6.2. Comparison with Previous Computer Vision Approaches

The recent literature on computer vision ergonomic assessment has demonstrated promising accuracy: [19] achieved 93% RULA classification accuracy using 3D pose estimation; ref. [20] reported 82–94% agreement between automated and expert RULA scoring. Recent comparative studies have validated 2D pose estimation against inertial measurement units for postural risk assessment in clinical settings [16,21]. However, these studies focused on snapshot accuracy assessing individual postures from images. This research advances beyond snapshot accuracy to address continuous temporal dynamics. The 95% correlation coefficient (Pearson’s r) between continuous risk evolution and alert time, combined with mean response latency of 42.1 ms, establishes the first demonstration of real-time proprioceptive feedback in ergonomic technology. This represents a categorical advance from precise assessment to active intervention.

### 6.3. Biomimetic Design as Competitive Advantage

Our system does not seek to directly replicate biological mechanisms, but rather implements a functional translation of the proprioceptive feedback loop. Unlike biological systems, which integrate complex multisensory signals (vestibular, cutaneous, muscular) and rely on adaptive neuromuscular learning, our approach focuses on isolating two key functionalities to emulate the protective response of the spinal reflex: continuous monitoring and immediate feedback latency (<50 ms). This difference is crucial: the system is a bio-inspired alert framework, not a physiological reproduction. Its purpose is to provide a technological substitute to improve situational awareness in industrial environments.

Biological inspiration driving this research provided three specific design innovations [22]:

- **Continuous Monitoring:** Instead of episodic evaluation, proprioceptive systems operate continuously. This architecture revealed that dynamic task execution creates risk peaks never detected by single-photo assessment, explaining why workers develop injuries despite “acceptable” static RULA scores.
- **Immediate Feedback:** Biological systems activate corrective response immediately upon hazard detection (spinal reflex latency < 100 ms). Our 42 ms system latency allows real-time worker adaptation: if a worker hears an alert while lowering a load, they can immediately adjust posture, preventing injury consolidation.
- **Multisensory Integration:** Biological feedback combines proprioceptive, vestibular, and visual information. Our multi-channel alert (audio + visual + optional haptic) replicates this integration, improving behavioral responsiveness versus single-channel alerts.

### 6.4. Limitations and Mitigation Strategies

Experimental validation was conducted on a cohort of healthy young adults (18–25 years). While this verified the technical latency and angular accuracy of the system, we recognize that this does not fully represent the demographic reality of the industrial workforce [1,2]. Older workers often have reduced joint range of motion and may employ compensatory movement strategies developed over years of experience to minimize fatigue. These movement patterns could affect the reliability of the posture algorithm and require study conditions different from those of a younger population. Therefore, although the current results demonstrate the feasibility of the technological tool, future field studies should include older participants with pre-existing musculoskeletal conditions to ensure the generalization of the system to real-world situations.

- **Sensitivity to Occlusion:** Markerless vision systems present challenges when body segments are occluded, such as when hands are obscured during grasping activities.
  To address occlusion in industrial applications, where machinery or personal protective equipment (PPE) can obstruct the camera’s view, two specific mitigation strategies are proposed for future development: (1) Implementation of a multi-camera fusion system, where data from orthogonal views are synthesized to cover blind spots. A monitoring cell is created with 2 or 3 synchronized cameras facing the worker. (2) Confidence thresholds, where the system is programmed to suppress risk alerts if MediaPipe’s visibility score for key points falls below 0.5.
- **Kinematic-Only Assessment:** Computer vision measures joint angles but cannot directly quantify force, grip quality, or muscle effort. RULA itself is only kinematic; integration with force-sensing gloves [23] or IMU sensors represents future improvement.
- **Sensitivity to Camera Positioning:** While 3D measurement eliminates 2D projection error, optimal accuracy still requires lateral positioning (side view). Frontal or oblique views introduce angle ambiguity. Practical implementation includes camera positioning guidelines.
- **Validity vs. efficacy:** It is important to distinguish between technical validation and ergonomic validity. This study confirms the geometric accuracy of the system (through depth detection) and processing speed. However, we have not cross-validated the risk scores with physiological measures such as electromyography (EMG) or optical motion capture. Therefore, although the system accurately detects kinematic deviations according to RULA thresholds, future studies are needed to confirm that these risk thresholds directly correlate with physiological muscle fatigue.
- **Task specificity:** The experimental protocol was restricted to a single standardized lifting task with a fixed load (6 kg) to ensure repeatability. We acknowledge that this simplification does not reflect the complexity of multi-task industrial environments involving repetitive cycles or varying loads.
- **Fixed joint parameters:** A notable limitation of vision-based assessment is the inability to reliably detect subtle rotational movements, specifically wrist twist and complex head rotation, due to self-occlusion. In this implementation, these components were fixed to default ’lowest risk’ values to prevent false positives. This approach inherently risks underestimating the total RULA score in tasks where wrist manipulation is the primary stressor.

### 6.5. Implications for Occupational Ergonomic Practice

The continuous application of the RULA method, originally designed for discrete observation, at a sampling frequency of 30 Hz introduces specific interpretation challenges. Traditional RULA identifies static risk postures; in contrast, continuous monitoring captures both the cumulative biomechanical load and the transient high-risk peaks associated with the dynamic phases of movement (acceleration/deceleration). Therefore, the “continuous RULA score” should be understood as a temporal profile of risk exposure. This profile extends beyond a simple sequence of high-frequency static assessments, highlighting dynamic kinematic deviations that are undetectable by conventional snapshot-based methods.

Current occupational ergonomics relies on periodic expert audits. Our system enables continuous monitoring with real-time feedback, a qualitatively different operational mode:

- **Immediate Intervention:** Instead of discovering postural risks during annual audits, workers receive real-time correction signals during task execution.
- **Behavioral Adaptation:** Repeated exposure to the alert allows proprioceptive learning; workers internalize safer movement patterns.
- **Objective Documentation:** Continuous data capture provides evidence-based justification for workstation redesign, rather than subjective ergonomist recommendations.
- **Scalable Implementation:** CPU-based processing and affordable RGB-D sensors allow implementation across multiple workstations.

### 6.6. User Experience Considerations

From a human–computer interaction (HCI) perspective, continuous auditory feedback introduces the risk of “alert fatigue.” As noted in previous studies of real-time feedback [24], if workers receive constant warnings, they may become desensitized or annoyed. Therefore, future iterations should implement adaptive feedback mechanisms, where alerts are triggered only by sustained risky behaviors (e.g., trigger only if RULA ≥ 5 is maintained for more than 3 s) rather than reacting to momentary high-threshold movements. Furthermore, switching from purely negative reinforcement (alarms) to positive reinforcement (gamification scores for “safe” shifts) could improve user acceptance and behavioral compliance.

## 7. Pseudocode and Software Implementation

The complete algorithm for real-time risk estimation is presented in Algorithm 1.
**Algorithm 1** Real-Time Risk Estimation Procedure**Input:** RGB-D Video Stream (Orbbec Femto Mega)**Output:** RULA Score, Augmented Visualization, Alert Signal  1:Initialize Orbbec Pipeline and MediaPipe Holistic Model  2:Initialize Stabilization Filters ($\alpha_{trunk}=0.8,\alpha_{arm}=0.7,...$)  3:**WHILE** is_running **DO**:  4:    Frame_RGB, Frame_Depth ← Capture Synchronized()  5:    Landmarks_3D ← MediaPipe.Process(Frame_RGB)  6:** **  7:    **IF** Landmarks_3D detected **THEN**:  8:        # 1. Laterality Determination  9:        Active_Side ← Compare_Depth(Left_Shoulder.z, Right_Shoulder.z)10:** **11:        # 2. Biomechanical Calculation (Vector)12:        Arm_Angle ← Calculate_3D_Angle(Shoulder_Elbow_Vector, Trunk_Vector)13:        Trunk_Angle ← Calculate_3D_Angle(Trunk_Vector, Vertical_Gravity_Vector)14:        Neck_Angle ← Calculate_3D_Angle(Shoulder_Ear_Vector, Trunk_Vector)15:** **16:        # 3. Filter Application17:        Smooth_Arm_Angle ← Stabilizer.update(Arm_Angle)18:        ... (repeat for all segments)19:** **20:        # 4. RULA Scoring Logic (Mapping to Tables)21:        Score_A ← Table_A(Score(Arm), Score(Forearm), Score(Wrist))22:        Score_B ← Table_B(Score(Neck), Score(Trunk), Score(Legs))23:        Final_Score ← Table_C(Score_A, Score_B)24:** **25:        # 5. Bio-inspired Feedback Mechanism26:        Emit_UI_Signal(Final_Score, Angles)27:        **IF** Final_Score ≥ 5 **AND** Current_Time - Last_Alert > 2.0s **THEN**:28:            Frequency ← 1000Hz (If Score < 7) OR 2500Hz (If Score = 7)29:            Generate_Auditory_Alert(Frequency)30:        **END IF**31:    **END IF**32:**END WHILE**

## 8. Conclusions

This research establishes proof-of-concept for bio-inspired ergonomic assessment systems that translate natural proprioceptive feedback principles into automated occupational monitoring technology. Key contributions include the following:

- **Continuous Monitoring Architecture:** Extension of previous computer vision approaches to implement continuous temporal assessment rather than snapshot-based assessment, demonstrating 62.5% critical risk prevalence during dynamic tasks versus 7.5% in static assessment.
- **Metrological Advance:** Three-dimensional vector-based angle calculation overcomes fundamental two-dimensional projection limitations documented in traditional RULA assessment.
- **Temporal Precision:** Correlation precision of 95% (95% CI: 0.91–0.97) and latency of 42.1±8.3 ms allow immediate alignment of postural feedback with natural spinal reflex timing.
- **Technical Validation:** Empirical demonstration of system feasibility for real-time occupational monitoring in controlled environments.
- **Biomimetic Design Framework:** Systematic translation of documented biological proprioceptive mechanisms into occupational technology provides a grounded basis for future development.
- **Practical Accessibility:** CPU-based processing and affordable RGB-D sensors allow potential industrial implementation without specialized laboratory infrastructure.

**Important Scope Clarification:** This work demonstrates technical feasibility and system performance validation. It does not provide evidence that continuous real-time feedback reduces workplace injury incidence. Translating “the alert system works” to “the system prevents injuries” requires prospective longitudinal intervention studies measuring injury rates before and after implementation in target occupational populations. Such studies represent essential future work but fall outside the scope of this technical validation.

**Limitations:** The study population (university students, 18 to 25 years) represents a narrow age range and limited occupational context. Results may not generalize to experienced manual workers with compensatory movement strategies, older workers, or industrial environments with sustained high-load tasks. The adapted RULA-based 3D scoring reflects modifications to fit 3D kinematics and may not be directly comparable to original 2D RULA tables without additional biomechanical validation.

The bio-inspired architecture (continuous monitoring, automatic response, objective measurement, immediate feedback) directly replicates mechanisms through which biological systems detect and correct postural deviation. By translating these natural design solutions into occupational technology, we establish a foundation for evidence-based development of bio-inspired workplace safety systems.

Future research directions include (1) multi-camera integration for occlusion mitigation; (2) incorporation of force sensing modality for comprehensive biomechanical assessment, potentially integrating bio-inspired soft robotic principles [25]; (3) machine learning-based postural prediction for anticipatory alert generation; (4) longitudinal field studies in target occupational populations (manufacturing, healthcare, construction) documenting injury reduction from continuous monitoring interventions; and (5) comparative cost-benefit analysis versus traditional ergonomic audit approaches.

This study validates the technical feasibility of the system. To test clinical efficacy (reduction in injuries), a future longitudinal study is required. This study would involve a controlled field trial over a period of 6 to 12 months in a high-risk facility, dividing workers into an intervention group (receiving real-time feedback) and a control group (standard practice). Key outcome indicators would include the incidence rate of reported musculoskeletal discomfort and sick leave (medical leave). Such a rigorous design is necessary to confirm whether the effectiveness of the tool translates into a statistically significant reduction in workplace injuries.

To bridge the gap between kinematic detection and physiological reality, future work should consider hybrid approaches. Recent research has demonstrated the value of combining depth vision with muscle–computer interfaces to interpret neuromuscular intent [13]. Additionally, implementing adaptive decision mechanisms, such as fuzzy logic systems, could refine how risk thresholds are handled under uncertainty, as explored in recent bio-inspired interface reviews [15]. 

## Figures and Tables

**Table 1:** Mapping of biological proprioceptive system to technical implementation.

Biological Component	Function	Technical Implementation
Proprioceptive receptors	Continuous posture sampling	MediaPipe Holistic at 30 FPS
Joint angle detection	3D orientation measurement	Vector-based angle calculation
Sensory–neural transmission	Latency <50 ms	Processing latency 18.3 ms (21.8 ms avg)
Spinal processing	Risk classification	RULA lookup table implementation
Motor correction command	Corrective signal generation	Audio–visual alert (<100 ms trigger)
Feedback loop closure	Behavioral adaptation	Continuous monitoring enables worker learning

**Table 2:** System workflow stages and technology.

Stage	Activity	Main Technology
I. 3D Capture	The optical sensor (Orbbec) captures RGB video and depth data.	Orbbec Femto Camera (RGB-D Sensor)
II. Pose Detection	The computer vision algorithm identifies the subject and extracts 33 key skeletal landmarks in 3D coordinates (X, Y, Z).	Google MediaPipe Holistic (BlazePose)
III. Pre-processing	A stability filter (Stabilizer) is applied to joint Z-coordinates to eliminate noise and camera vibrations, achieving stable data.	Exponential Smoothing Algorithm
IV. Biomechanical Calculation	Stable 3D coordinates are used to calculate joint angles using the Dot Product formula.	3D Vector Algebra (Dot Product)
V. Ergonomic Evaluation	Calculated angles are input into the rules engine (RULA tables) to obtain the risk score (Score 1 to 7).	RULA Rules Engine (Tables A, B, C)
VI. Output and Feedback	Score and angles are displayed on the PC interface, activating an auditory alert if risk is high.	Graphical Interface (PyQt6) and Audio Signal (Winsound)

**Table 3:** Technical specifications and operational parameters of Orbbec Femto Mega.

Parameter	Specification	Selection Justification
Sensor Technology	Time of Flight (ToF)	Superior stability vs. structured light
RGB Resolution	1280 × 720 @ 30 FPS	High-resolution pose context
Depth Resolution	640 × 576 @ 30 FPS (NFOV)	Adequate for joint localization
Depth Measurement Range	0.2–5.0 m	Standard occupational distance
Depth Accuracy	2% @ 1 m	Sub-degree angle uncertainty
Alignment Mode	Hardware (HW_MODE)	Real-time color-depth synchronization
Field of View	70° (horizontal)	Typical workstation geometry
Power Requirement	USB 3.0 (<500 mA)	Field deployment without external PSU
Integrated SDK	pyorbbecsdk (Python)	Direct integration with analysis pipeline

**Table 4:** Demographic and anthropometric characteristics of participants (n=40).

Characteristic	Mean	SD	Range
Age	21.3 years	1.8	18–25
Sex	65% Female, 35% Male		
Height	171.2 cm	8.3	155–192
Body Mass	71.4 kg	12.6	52–98
Body Mass Index (BMI)	24.3 kg/m^2^	3.9	18.5–32.1

**Table 5:** Comparison of static resting posture versus dynamic load lifting task (N=40). Note: *n* represents the subset of participants exhibiting critical risk in each condition (subset count), distinct from experimental repetitions.

Evaluation Condition	Participants at Critical Risk (*n*, %)	Mean RULA	Interpretation
Static Rest (baseline)	n=3 (7.5%)	2.1±0.8	Posture largely acceptable
Dynamic Load Lifting	n=25 (62.5%)	5.2±1.1	Majority exhibit critical risk
**Difference**	**+22 participants (+55.0%)**	**3.1 points**	**8-fold increase in risk prevalence**

**Table 6:** RULA score distribution and action-level classification (n=40).

RULA Score	Action Level	Participants	Percentage	Interpretation
1–2	Acceptable	0	0%	No postural risk
3	Investigate	6	15%	Possible intervention needed
4	Investigate	12	30%	Task-specific investigation
5–6	Adopt Changes Soon	20	50%	Changes required; intervention likely needed
7	Urgent Changes	2	5%	Serious concern; urgent intervention
**Critical (*RULA* ≥ 5)**		**22**	**55%**	**Requires immediate action**

**Table 7:** Real-Time processing performance and sensor stability metrics.

Performance Parameter	Mean ± SD	Unit
Processing Frame Rate	29.8±0.2	FPS
Color Frame Capture Rate	30.0±0.0	FPS
Depth Frame Capture Rate	29.9±0.1	FPS
Landmark Detection Success Rate	97.2±1.8	%
3D Coordinate Jitter (w/o smoothing)	2.8±0.6	pixels
3D Coordinate Jitter (w/smoothing)	0.6±0.2	pixels
Joint Angle Stability (SD within 2 s window)	0.34±0.15	degrees
RULA Score Recalculation Latency	18.3±3.2	ms
Alert Trigger Response Time	42.1±8.3	ms

**Table 8:** Alert system validation: correlation between calculated risk and alert activation.

Metric	Mean	SD	Range
Pearson Correlation (Risk ↔ Alert)	0.95	0.04	0.88–0.99
Participants with r>0.90	38		95%
Alert Activation Count per Participant	8.4	5.9	1–24
Time from Risk Onset to Alert Trigger	42.1	8.3	31–58 ms
False Alarm Rate (score <5 triggering alert)	1.3	0.8	%
Missed Alert Rate (score >5 no alert)	0.5	0.4	%

**Table 9:** Anatomical segment contribution to critical postural risk (RULA≥5).

Anatomical Segments	Body Segment	Participants Involved	%	Mean Angle/Risk Category
Trunk Flexion	Shoulders to Hips	35 of 40	87.5%	48.2° ± 11.6° /Primary
Arm Abduction	Shoulder–Elbow–Hip	29 of 40	72.5%	92.3° ± 22.1° /Primary
Arm Flexion	Shoulder–Elbow–Hip	27 of 40	67.5%	87.4° ± 15.3° /Secondary
Cervical Flexion	Ears–Shoulders	22 of 40	55.0%	44.1° ± 9.8° /Secondary
Forearm Sup/Pron	Elbow–Wrist	10 of 40	25.0%	38.5° ± 12.4° /Tertiary

**Table 10:** Individual participant RULA assessment results: Representative sample of 20 of 40 participants.

ID	Action Level	Max RULA	Critical Segments	Static RULA
P001	Adopt Changes	5	Trunk 34°, Arm 133°	2 (Acceptable)
P002	Adopt Changes	5	Trunk 65°, Neck 42°	2 (Acceptable)
P003	Investigate	3	Arm 58°	2 (Acceptable)
P004	Adopt Changes	5	Neck 53°, Arm 105°	2 (Acceptable)
P005	Investigate	4	Trunk 42°, Arm 99°	2 (Acceptable)
P006	Adopt Changes	5	Trunk 46°, Neck 53°	2 (Acceptable)
P007	Adopt Changes	5	Arm 107°, Trunk 39°	3 (Investigate)
P008	Adopt Changes	5	Trunk 62°, Neck 62°	3 (Investigate)
P009	Adopt Changes	5	Arm 97°, Trunk 49°	3 (Investigate)
P010	Adopt Changes	5	Neck 44°, Trunk 31°	2 (Acceptable)
P011	Adopt Changes	5	Trunk 60°, Arm 52°	2 (Acceptable)
P012	Adopt Changes	5	Trunk 54°, Neck 54°	3 (Investigate)
P013	Adopt Changes	5	Arm 133°, Trunk 59°	2 (Acceptable)
P014	Adopt Changes	6	Arm 172° (Extreme), Trunk 55°	2 (Acceptable)
P015	Adopt Changes	5	Trunk 30°, Neck 30°	3 (Investigate)
P016	Investigate	4	Arm 71°, Trunk 25°	2 (Acceptable)
P017	Adopt Changes	5	Trunk 52°, Neck 48°	2 (Acceptable)
P018	Investigate	3	Forearm 42°	2 (Acceptable)
P019	Adopt Changes	5	Arm 88°, Trunk 44°	2 (Acceptable)
P020	Investigate	4	Neck 35°, Arm 68°	2 (Acceptable)

## Data Availability

Extended participant-level data for the 40 subjects, raw 3D coordinate sequences, detailed temporal alert activation logs, video demonstrations of system operation, and calibration protocols are available upon request, please contact the corresponding author to access the full dataset or source code.
